# Negative correlation between cardiometabolic index and testosterone in male adults

**DOI:** 10.3389/fendo.2024.1447230

**Published:** 2024-12-11

**Authors:** Jing Xu, Yue-Chun Li

**Affiliations:** ^1^ Department of Endocrinology, The Second Affiliated Hospital and Yuying Children’s Hospital of Wenzhou Medical University, Wenzhou, China; ^2^ Department of Cardiology, The Second Affiliated Hospital and Yuying Children’s Hospital of Wenzhou Medical University, Wenzhou, China

**Keywords:** cardiometabolic index, testosterone deficiency, insulin resistance, diabetes mellitus, obesity

## Abstract

**Background:**

Insulin resistance (IR) is closely correlated with a deficiency or decrease of testosterone levels in males. Cardiometabolic index (CMI) is correlated with various diseases correlated with IR. The primary objective of this study is to explore the correlation between CMI and testosterone levels in male adults.

**Methods:**

Data from the National Health and Nutrition Examination Survey (NHANES) during the period from 2013 to 2020 were analyzed through a cross-sectional design. CMI was calculated by multiplying waist-to-height ratio (WHtR) with the triglyceride-to-high-density lipoprotein cholesterol ratio (TG/HDL-C).

**Results:**

A total of 5012 subjects were included in the final analysis. After controlling confounding variables, multiple linear regression analysis indicated an independent negative correlation between CMI and testosterone levels (β= -6.40, 95% CI: -8.95, -3.86, P<0.001) through the. In addition, a negative non-linear correlation was also found between CMI and testosterone (P<0.05), with CMI’s inflection point as 0.73. Subgroup analyses indicated a more significant negative correlation among those with normal weight and the elderly (*p*< 0.05 for all interactions). The area under the ROC curve (AUC) of CMI (AUC =0.724, 95% CI: 0.709–0.740) was the largest compared with those of TG/HDL and WHtR.

**Conclusion:**

Elevated CMI is significantly and negatively correlated with testosterone in male adults.

## Introduction

As a crucial male sex hormone produced by Leydig cells, testosterone plays a vital role in various physiological processes in males, such as cardiovascular health, sexual function, bone density, metabolism and cognitive function (1–3). The deficiency or decrease of serum testosterone levels in males can lead to the dysfunction in various organs. And low testosterone level may also contribute to or exacerbate metabolic conditions such as osteoporosis and depression, known as male hypogonadism (4) or testosterone deficiency syndrome (5–8), alongside diminished erectile dysfunction and libido. Testosterone deficiency is a prevalent disorder affecting approximately 7% of males aged over 50 years old, with its prevalence rising with age. It is projected that the prevalence of this condition will escalate with the increasing average lifespan in the coming decades (9). The prevalence of testosterone deficiency has emerged as a growing concern global.

IR is recognized as a key contributing factor in the pathogenesis of cardiometabolic diseases, with hypogonadism being frequently observed in individuals with metabolic comorbidities such as diabetes mellitus and obesity (10). Multiple studies have underscored the strong correlation between testosterone deficiency and IR (10–14). Souteiro et al. found that IR is the predominant risk factor for low testosterone levels in males with obesity, and some individuals experiencing testosterone deficiency demonstrating a higher IR compared to those with mild diabetes mellitus (11). Furthermore, a deficiency or decrease in testosterone has been correlated with the onset of metabolic disorders, such as elevated visceral lipids and IR (15). Additionally, the reciprocal correlation between hypogonadism and metabolic disorders has been confirmed in instances of late-onset and functional hypogonadism (16). Consequently, the investigation on the correlation between IR and male testosterone has become a prominent field for studying.

The intricacy, protracted process, and restricted utility of conventional IR assessments, such as HOMA-IR, have necessitated the exploration of alternative methodologies. The scholar works have been extensively examined the correlation between dyslipidemia, obesity and IR (17–19). Specifically, WHtR and TG/HDL-C has emerged as a valuable indicator for effectively evaluating IR. Derived from the multiplication of WHtR by TG/HDL-C, CMI was first introduced by Ichiro Wakabayashi in 2015 as a mean of evaluating the susceptibility to cardiometabolic disorders, T2DM, and IR (20–22). Currently, there are few studies exploring the potential correlation between CMI and testosterone.

Therefore, this study aims to explore the correlation between CMI and testosterone in male adults through a nationally representative sample on American adults, as well as assessing the predictive utility of CMI in detecting testosterone deficiency.

## Methods

### Subjects

For a cross-sectional study, data were obtained from NHANES, a research program sponsored by the Centers for Disease Control and Prevention (CDC) to assess the health conditions of American citizens during the period from 2013 to 2020. Due to the global outbreak of COVID-19, NHANES has been suspended from 2020 and beyond. Additionally, the samples were highly representative of the national population of US as NHANES applied a stratified multi-stage sampling method. All the data adopted from NHANES could be obtained at https://www.cdc.gov/nchs/nhanes/.

**Figure 1:**
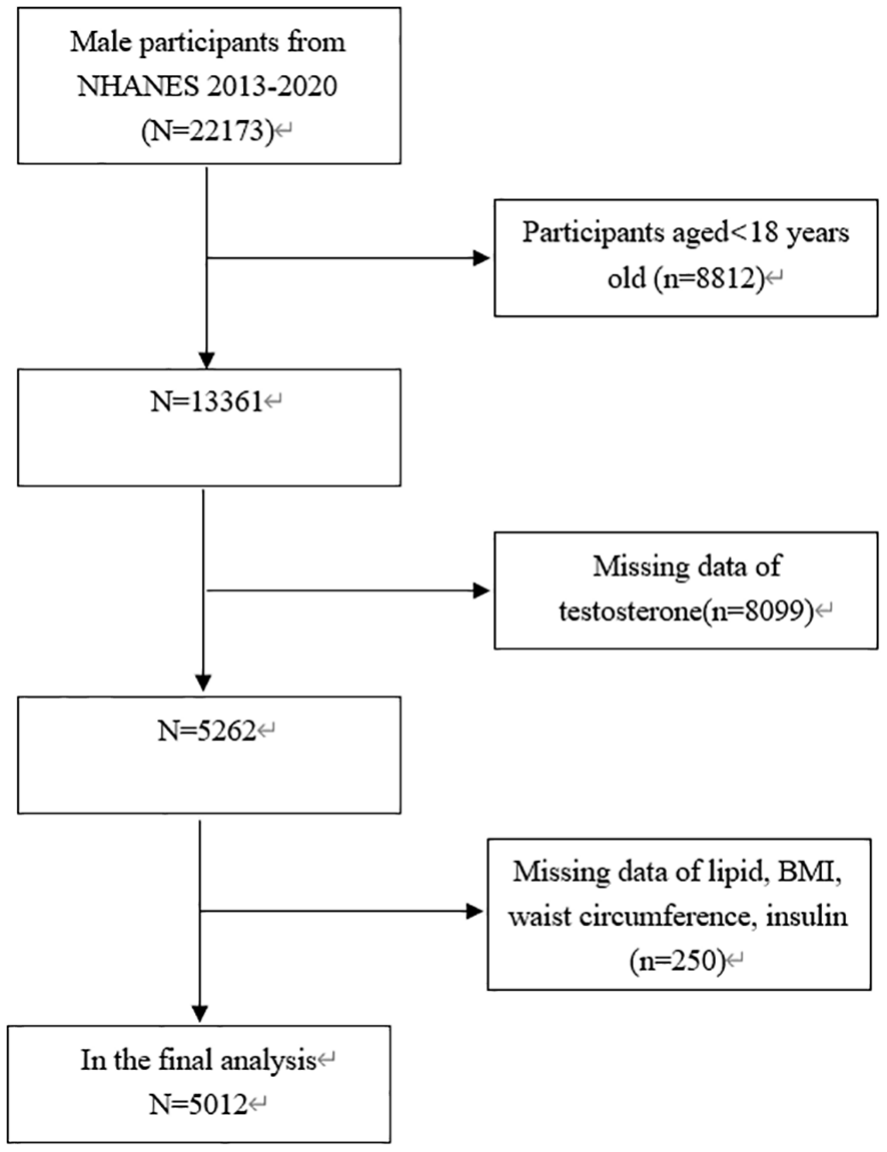
Flowchart of the sample selection from the 2013-2020 NHANES.

This study focused exclusively on males aged 18 years old and above (n =22173). In addition, 17161 subjects were eliminated due to missing data on testosterone (n = 8099) or BMI, lipid, waist circumference and insulin data (n = 250). Consequently, 5012 subjects aged from 18 to 80 years old were involved in the final analysis (**Figure 1**).

NHANES was approved from NCHS Ethics Review Board for its implementation, and written informed consent has been received from all subjects (23).

### Anthropometric measurements

Demographic and lifestyle information was collected via household interview questionnaires administered by highly trained medical personnel. Anthropometric indices and biochemical parameters were acquired through medical examinations and subsequent laboratory analyses conducted at the Mobile Examination Centre (MEC). Data collected at enrollment included the information on history of diabetes mellitus, education level, physical activities, race, height, weight, blood pressure and waist circumference (WC), dietary intake factors, encompassing energy and fat. All the participants completed 24-hour dietary recalls on the mean consumption rates derived from these two recalls, obesity as BMI≥30kg/m^2^, and normal weight or overweight as BMI<30kg/m^2^.

TC, FINS, HbA1c, LDL-C, ALT, UA, TG, estradiol (E2), AST, creatinine, albumin, sex hormone binding globulin (SHBG), HDL-C, testosterone and FPG were collected with blood samples. Less than 3% of values were missed in total. Multiple imputation was performed for missing values. Detailed measurement methodology and data acquisition for each variable can be accessed at www.cdc.gov/nchs/nhanes. As defined in the American Urological Association Guidelines, testosterone deficiency is characterized as total testosterone levels below 300 ng/dL (24).

IR was assessed with HOMA-IR formula, calculated by multiplying FPG in mmol/L by FINS in IU/L, and then dividing by 22.5 (25). The formula for calculating CMI was expressed as [WC in cm/height in cm] *[TG in mmol/L/HDL-C in mmol/L] (20).

### Statistical analysis

Subjects were categorized into quartiles based on CMI levels (Q1: ≤0.373; Q2: 0.373-0.699; Q3: 0.699-1.347; Q4: ≥1.347). The normality of continuous variables were assessed by presenting them as either median and interquartile range or mean ± SD in this study. Disparities among quartile groups were evaluated through Kruskal-Wallis H test for continuous variables and chi-square tests for categorical variables. The correlation between CMI and metabolic risk factors was examined through Spearman’s correlation analysis. And the correlation between CMI and testosterone was explored through a regression model analysis, with β values and 95% CI as indicators. Meanwhile, three models were utilized in the study: Model I without any adjustment, Model II with adjustments for race and age, and Model III with adjustments for DBP, age, SHBG, race, SBP, diabetes mellitus, E2, HOMA-IR, AST, moderate physical activities, HbA1c, serum uric, ALT, serum creatinine, albumin, education level, total energy and total fat intake. Subgroup analyses were conducted to stratify patients according to diabetes mellitus status, age, education level, race, moderate physical activities and BMI. Additionally, generalized additive models and smooth curve fitting were utilized to explore potential nonlinear correlation between CMI and testosterone. The diagnostic utility of CMI, TG/HDL-C, and WHtR in detecting testosterone deficiency was evaluated through ROC curve analysis. Statistical analyses were performed with EmpowerStats software and R, with statistical significance at *p*< 0.05.

## Results

### Baseline characteristics of subjects

A total of 5012 subjects ranging from 18 to 80 years old were included in this study, with a prevalence of testosterone deficiency at 25.7%. The demographic characteristics of the subjects stratified by CMI quartiles are presented in **Table 1**. Subjects in the highest CMI quartile exhibited a higher prevalence of diabetes mellitus, testosterone deficiency and elevated AST, weight, uric acid, BMI, TG, waist circumference, ALT, hemoglobin A1c, TC, LDL-C, total energy and total fat intake, compared to those in the lowest quartile. Conversely, subjects in the highest quartile had lower HDL-C, SHBG and moderate physical activities (*p*<0.01) (**Table 1**).

**Table 1:** Weighted characteristics of the study population based on CMI quartiles.

Characteristic	Q1	Q2	Q3	Q4	P value
N	1253	1253	1253	1253	
Age, years	44.1 ± 19.8	47.2 ± 18.9	49.0 ± 17.5	49.3 ± 16.2	<0.001
Race, n%					<0.001
Mexican American	129 (10.3)	177 (14.1)	224 (17.9)	266 (21.2)	
Other Hispanic	400 (31.9)	279 (22.3)	196 (15.6)	121 (9.7)	
Non-Hispanic White	450 (35.9)	477 (38.1)	497 (39.7)	520 (41.5)	
Non-Hispanic Black	88 (7)	123 (9.8)	152 (12.1)	141 (11.3)	
Other Race	186 (14.8)	197 (15.7)	184 (14.7)	205 (16.4)	
Moderate physical activities, n%					<0.001
Yes	574 (45.8)	543 (43.4)	517 (41.3)	477 (38.1)	
No	679 (54.2)	709 (56.6)	735 (58.7)	776 (61.9)	
Diabetes, n%					<0.001
Yes	86 (7)	139 (11.3)	200 (16.3)	251 (20.9)	
No	1146 (93)	1087 (88.7)	1024 (83.7)	952 (79.1)	
Education level, n%					0.041
Less than high school	889 (78.9)	930 (78.5)	923 (76.4)	920 (74.6)	
High school or above	238 (21.1)	254 (21.5)	285 (23.6)	314 (25.4)	
Testosterone deficiency, n%	125 (10)	222 (17.7)	369 (29.4)	571 (45.6)	<0.001
Body mass index, Kg/m^2^	24.8 ± 4.5	27.7 ± 5.6	30.0 ± 6.0	31.7 ± 5.9	<0.001
Waist circumference, cm	89.5 ± 12.9	98.3 ± 14.5	104.7 ± 15.1	109.3 ± 14.9	<0.001
Systolic blood pressure, mmHg	123.2 ± 16.5	124.4 ± 17.8	125.5 ± 16.3	128.3 ± 16.3	<0.001
Diastolic blood pressure, mmHg	68.2 ± 12.2	69.8 ± 13.5	71.0 ± 13.5	73.3 ± 13.5	<0.001
Hemoglobin A1c, mmol/L	5.48 ± 0.71	5.63 ± 0.89	5.84 ± 1.08	6.15 ± 1.43	<0.001
FPG, mmol/L	5.5 (5.1, 5.9)	5.7 (5.3, 6.2)	5.9 (5.4, 6.7)	6.0 (5.5, 7.1)	<0.001
FINS, uU/mL	6.1 (4.0, 8.7)	8.4 (5.7, 13.2)	12.3 (8.8, 18.1)	16.3 (10.8, 28.0)	<0.001
HOMA-IR	1.49 (0.98, 2.21)	2.20 (1.46, 3.47)	3.35 (2.33, 5.08)	4.74 (2.93, 8.92)	<0.001
ALT, U/L	24.6 ± 18.5	27.1 ± 22.4	29.4 ± 15.3	35.7 ± 25.0	<0.001
AST, U/L	27.7 ± 30.4	26.6 ± 15.6	26.9 ± 10.8	29.2 ± 14.3	0.003
Testosterone, ng/dL	527.5 ± 202.5	450.0 ± 173.6	385.6 ± 173.1	329.8 ± 137.4	<0.001
E2, pg/ml	25.8 ± 10.4	25.8 ± 10.0	24.3 ± 9.9	23.8 ± 9.0	0.098
SHBG, ug/L	47.4 (33.9, 66.2)	40.1 (29.0, 57.2)	35.5 (25.4, 49.5)	30.7 (21.5, 42.4)	<0.001
Albumin, g/dl	4.43 ± 0.34	4.40 ± 0.34	4.38 ± 0.32	4.37 ± 0.31	<0.001
Creatinine, umol/L	90.0 ± 59.6	89.7 ± 29.2	89.4 ± 28.8	90.3 ± 44.3	0.956
Uric acid, umol/L	334.0 ± 70.8	353.8 ± 71.8	368.4 ± 78.2	377.7 ± 80.3	<0.001
Total cholesterol, mmol/L	4.53 ± 0.95	4.71 ± 1.02	4.90 ± 1.06	5.24 ± 1.24	<0.001
Triglycerides, mmol/L	0.71 (0.58, 0.89)	1.16 (0.99, 1.38)	1.81 (1.52, 2.13)	3.30 (2.58, 4.31)	<0.001
HDL-cholesterol, mmol/L	1.64 ± 0.41	1.31 ± 0.25	1.13 ± 0.21	0.93 ± 0.19	<0.001
LDL-cholesterol, mmol/L	2.58 ± 0.80	2.93 ± 0.91	3.07 ± 0.97	3.00 ± 1.00	<0.001
Energy intake, kcal/d	2377.6 ± 892.4	2230.9 ± 815.4	2332.1 ± 883.6	2350.7 ± 921.9	0.001
Fat intake, g/d	91.4 ± 41.7	85.0 ± 38.4	89.3 ± 41.5	90.6 ± 43.3	0.003
CMI	0.25 (0.18, 0.31)	0.51 (0.44, 0.59)	0.96 (0.82, 1.14)	2.11 (1.63, 3.05)	<0.001

P<0.05 was deemed significant. FPG, fasting plasma glucose; FINS, fasting insulin; HOMA, homeostatic model assessment of insulin resistance; ALT, alanine aminotransferase; AST, aspartate aminotransaminase; E2, estradiol; SHBG, sex hormone binding globulin.

### Correlation between CMI and clinical and biochemical parameters

According to the Spearman correlation analysis, CMI was positively correlated with DBP, BMI, HbA1c, WC, SBP, FINS, FPG, TC, HOMA-IR and testosterone, as indicated in **Table 2** (all *p*<0.001).

**Table 2:** Spearmen’s correlation of CMI levels with clinical and biochemical parameters.

Variable	CMI	
	r	P
BMI	0.492	<0.001
WC	0.501	<0.001
SBP	0.129	<0.001
DBP	0.162	<0.001
HbA1c	0.255	<0.001
TC	0.244	<0.001
LDL-C	0.211	<0.001
FPG	0.286	<0.001
FINS	0.539	<0.001
HOMA-IR	0.556	<0.001
Testosterone	-0.443	<0.001

BMI, body mass index; WC, waist circumference; SBP, systolic blood pressure; DBP, diastolic blood pressure; HbA1c, glycosylated hemoglobin; TC, total cholesterol; LDL-C, Low density lipoprotein cholesterol; FPG, fasting plasma glucose; FINS, fasting insulin; HOMA, homeostatic model assessment of insulin resistance.

### Linear correlation between CMI and testosterone

β values and 95% CI for CMI and testosterone in various models are shown in **Table 3**. The findings suggest a significant and independent negative correlation between CMI and testosterone across different adjusted models (Model I, β= -33.81, 95% CI: -37.23, -30.39; Model II, β= -32.84, 95% CI: -36.24, -29.44; Model III, β= -6.40, 95% CI: -8.95, -3.86; all p < 0.01). Specifically, testosterone levels in Model III were significantly lower in the Q2, Q3, and Q4 groups compared to that in the Q1 group after adjusting for confounding variables.

**Table 3:** Multivariate regression analysis of CMI with testosterone.

	Model1 β (95% CI) P value	Model2 β (95% CI) P value	Model3 β (95% CI) P value
CMI index	-33.81 (-37.23, -30.39), <0.001	-32.84 (-36.24, -29.44), <0.001	-6.40 (-8.95, -3.86), <0.001
CMI quartile			
Q1	Reference	Reference	Reference
Q2	-77.54 (-91.1, -63.97), <0.001	-74.02 (-87.52, -60.52), <0.001	-19.91 (-34.38, -5.44), <0.001
Q3	-141.93 (-155.49, -128.36), <0.001	-136.49 (-150.08, -122.90), <0.001	-33.17 (-49.02, -17.32), <0.001
Q4	-197.71 (-211.27, -184.14), <0.001	-192.08 (-205.71, -178.44), <0.001	-45.83 (-64.45, -27.21), <0.001
P for trend	<0.001	<0.001	<0.001

Model I: None covariates were adjusted; Model II: age and race were adjusted; Model III: age, race, SBP, DBP, HOMA-IR, HbA1c, E2, SHBG, ALT, AST, serum creatinine, serum uric, albumin, diabetes, moderate physical activities, education level, total energy and total fat intake were adjusted.

### Subgroup analysis

To assess the impact of subgroups on the correlation between CMI and testosterone levels, subgroup analyses were conducted on race, age, diabetes mellitus, BMI, education level and moderate physical activities. As shown in **Table 4**, the negative correlation between CMI and testosterone levels was significantly affected by age and BMI (*p* < 0.05 for all interactions). The correlation between CMI and testosterone was stronger in subjects with normal weight and those aged 60 years old or more.

**Table 4:** Association between CMI and testosterone stratified by age, race, BMI, diabetes, moderate activities and Education level.

	β (95%CI) p value	P for interaction
Stratified by age		<0.001
Age<60 years old	-5.73 (-8.61, -2.85), <0.001	
Age≥60 years old	-7.11 (-12.14, -2.07), 0.006	
Race		0.307
Mexican American	-11.93 (-17.69, -6.18), <0.001	
Other Hispanic	-2.02 (-8.12, 4.08), 0.515	
Non-Hispanic White	-5.05 (-8.96, -1.13), 0.012	
Non-Hispanic Black	-15.71 (-26.07, -5.36), <0.001	
Other Race	-5.25 (-11.03, -0.54), 0.045	
Stratified by BMI		<0.001
BMI<30kg/m^2^	-12.40 (-16.70, -8.11), <0.001	
BMI≥30kg/m^2^	-4.66 (-7.67, -1.66), 0.002	
Stratified by diabetes		0.062
Yes	-7.47 (-13.11, -1.82), 0.010	
No	-6.49 (-9.35, -3.63), <0.001	
Stratified by moderate activities		0.586
Yes	-6.12 (-10.19, -2.04), 0.003	
No	-6.24 (-9.50, -2.97), <0.001	
Stratified by education level		0.765
Less than high school	-4.84 (-9.49, -0.18), 0.042	
High school or above	-6.95 (-10.00, -3.90), <0.001	

Age, race, diabetes, moderate activities, education level (not adjusted for in the subgroup analyses), SBP, DBP, total energy intake, HOMA-IR, HbA1c, E2, SHBG, ALT, AST, serum creatinine, serum uric, albumin, total energy and total fat intake were adjusted.

### Non-linearity and threshold effect analysis between CMI and testosterone

**Figure 2:**
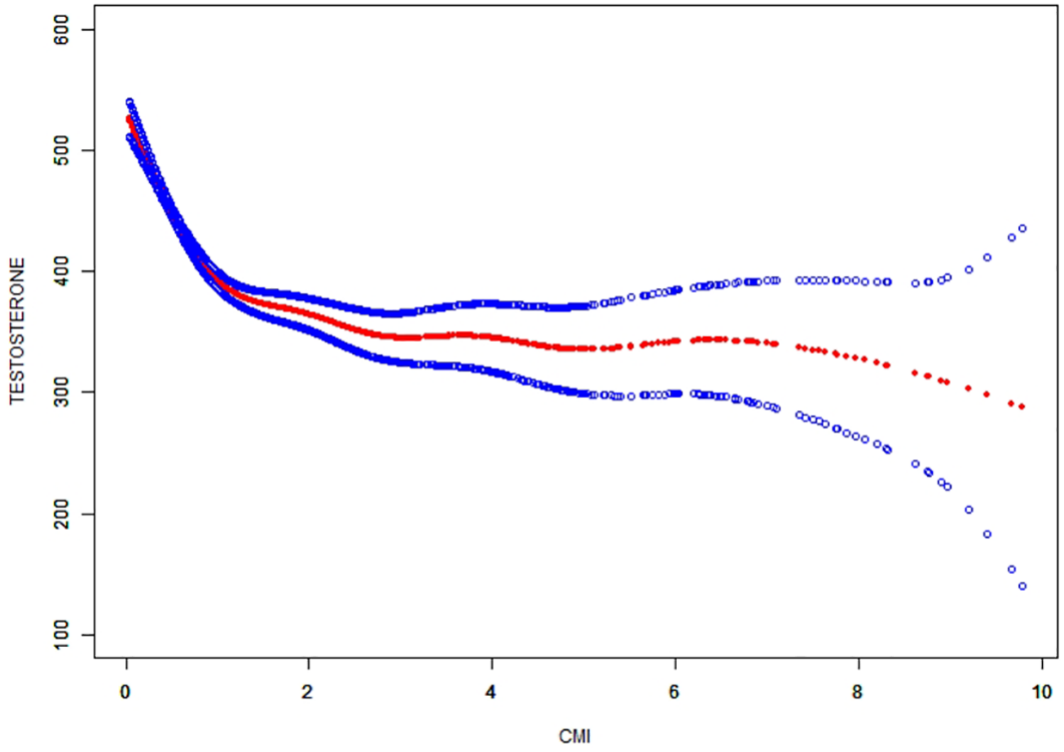
The smooth curve fit for the association between CMI and prevalence of testosterone.

**Figure 3:**
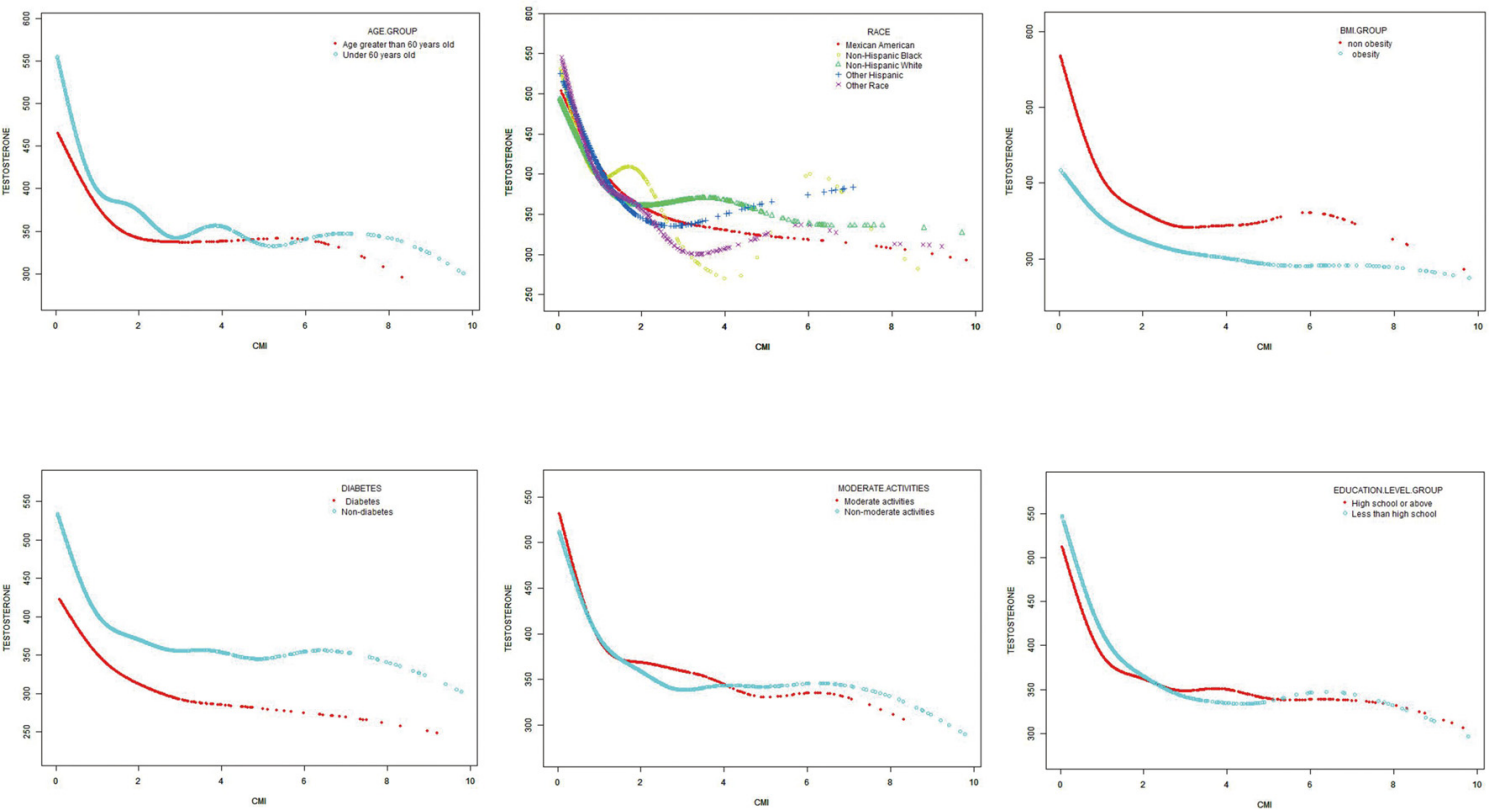
Subgroups analysis for the association between CMI and prevalence of testosterone by age, race, BMI, diabetes, moderate physical activities and education level.

The non-linear correlation and saturation effect between CMI and testosterone have been illustrated with smooth curve fitting techniques as depicted in **Figures 2**, **3**. In the subjects, a negative correlation was observed in the non-linear correlation, with inflection points at 0.73 (as shown in **Table 5**). Below the threshold of 0.73, there was a significant effect value of -209.16, while the value dropped to -14.82 when the measurement value of CMI exceeded 0.73.

**Table 5:** Threshold effect analysis of CMI on testosterone using the two-piecewise linear regression model.

UHR	Adjusted β (95% CI) P value
Fitting by the standard linear model	29.08 (-33.79, 24.37), <0.001
Fitting by the two-piecewise linear model	
Inflection point	0.73
CMI<0.73	-209.16(-238.41, -179.91), <0.001
CMI>0.73	-14.82 (-19.98, -9.66), <0.001
Log likelihood ratio	<0.001

### The predictive value of CMI for testosterone deficiency

**Figure 4:**
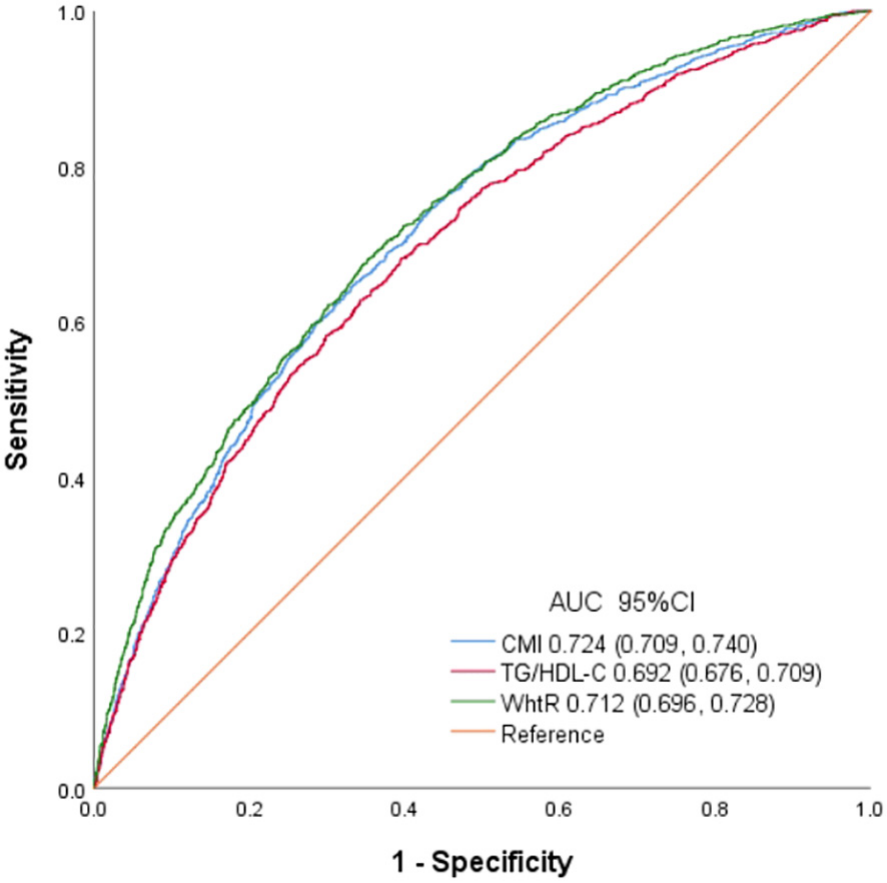
Receiver operating characteristic curves of CMI, TG/HDL and WhtR to identify testosterone deficiency.

ROC of CMI, TG/HDL and WHtR to diagnose testosterone deficiency is shown in **Figure 4**. AUC for CMI in the ROC analysis was found to be 0.724 (95% CI: 0.709–0.740), significantly higher than that for WHtR and TG/HDL (P < 0.001), with a sensitivity of 60.1%, a specificity of 71.3%, and a cutoff of 0.239.

## Discussion

The results showed that high CMI in American male adults is negatively correlated with serum testosterone levels. Subgroup analyses and interaction assessments showed a stronger correlation in the subjects with normal weight and those aged over 60 years old. In addition, the findings revealed a non-linear correlation between CMI and testosterone levels, with a notable inflection of CMI at 0.73. Furthermore, CMI had a superior diagnostic accuracy for testosterone deficiency compared to TG/HDL-C and WHtR alone.

CMI serves as an innovative clinical marker integrating the measurements of WHtR and TG/HDL-C. It was first discovered by Wakabayashi et al. in 2015 that CMI is of great significance in evaluating diabetes mellitus (20). Further research was conducted based on this discovery, revealing a significant correlation between CMI and IR related diseases such as kidney disease, cardiovascular disease, stroke and hypertension (26–29). suggesting that CMI may serve as a valuable indicator for metabolic diseases. However, there is a lack of data on the correlation between testosterone and CMI. This study is the first to effectively establish the correlation between CMI and testosterone in American male adults, utilizing a large sample size.

The potential risk factors linked to a deficiency or decrease in testosterone levels include advanced aging, obesity, hyperlipidemia and diabetes mellitus. IR plays a critical role in metabolic disorders, which is considered as a significant independent predictor of testosterone deficiency or decrease. Recent studies have confirmed a reciprocal correlation between IR and testosterone deficiency. IR can lower testosterone levels, leading to further development of obesity and IR (16). Tsai et al. conducted a study examining testosterone levels in 221 middle-aged males without diabetes mellitus. Multivariate analysis revealed a significant negative correlation between testosterone levels and C-peptide, insulin, and HOMA-IR (30). Additionally, a separate study involving 2361 Chinese males aged 20-73 years old identified a negative correlation between total testosterone levels and Mets (31). Similar findings were found in a study specifically focused on males in Taiwan (32). According to this study, there was a significant positive correlation between CMI and HOMA-IR, which is the key driving factor for testosterone decrease.

The distribution of body fat accumulation has a significant impact on the occurrence of IR and testosterone deficiency (5, 33–35). Previous studies have consistently demonstrated a strong correlation between traditional obesity indicators, such as WC and WHtR, and decreased testosterone levels (34, 35). TG/HDL-C has been extensively researched as a reliable and straightforward indicator of IR and testosterone deficiency in relation to T2DM (9, 36–38). Combining TG/HDL-C and WHtR, CMI can comprehensively evaluate dyslipidemia and obesity, making it a valuable tool for identifying testosterone deficiency. The findings as illustrated in **Figure 4** demonstrated that CMI is superior to TG/HDL and WHtR in terms of AUC, indicating its superior ability to detect testosterone deficiency. These results suggested that CMI may be suitable for evaluating testosterone deficiency in clinical settings.

Previous studies have reported that CMI is correlated with various diseases related to IR (20, 25, 39). Ichiro Wakabayashi et al. conducted a cohort survey of 10,196 individuals underwent a health assessment, revealing a significant positive correlation between elevated CMI values and the presence of hyperglycemia and the increased risk of diabetes mellitus (20). Additionally, Zou et al. determined that CMI was a reliable predictor of NAFLD within the general Japanese population (39). Furthermore, Luo et al. identified a positive correlation between high CMI values and the development of cardiovascular disease in patients with hypertension and obstructive sleep apnea (40). Another study by Xu et al. demonstrated a significant positive correlation between CMI and IR in patients with T2DM (25). The findings align with the conclusions drawn from the aforementioned studies. A significant correlation was observed between CMI and various factors, including BMI, HbA1c, WC, SBP, TC, DBP, HOMAI-IR, suggesting a potential value for CMI in future clinical applications.

The influence of established risk factors on the correlation between CMI and testosterone levels was further explored through stratified analyses. Studies have shown that the correlation is more obvious among subjects with normal weight and the elderly. It is well known that aging has been viewed as a risk factor for the development of hypogonadism (41, 42). Individuals with normal BMI and high body fat have a higher correlation with metabolic syndrome and IR (43). It is important to note that testosterone decrease is often overlooked in the subjects, emphasizing the importance of considering CMI as a factor for identifying testosterone decrease, particularly in this population.

The specific mechanism by which CMI leads to a decrease in male testosterone is still unclear. However, abnormal lipid metabolism and obesity may explain these results in CMI patients. Firstly, CMI functions as an indicator of abdominal obesity, which can directly influence testosterone levels. An increase in obesity is correlated with elevated levels of leptin, a hormone that stimulates the release of luteinizing hormone (LH) via hypothalamic gonadotropin-releasing hormone (GnRH) neurons. However, hypothalamus may develop resistance to leptin’s effects, leading to diminished feedback stimulation of testosterone production (44, 45). Furthermore, adipocytes prevalent in individuals with obesity exhibit elevated expression of aromatase, which are capable of producing inflammatory cytokines. Aromatase facilitates the conversion of testosterone into estradiol, consequently reducing circulating androgen levels. In addition, the increased estrogen levels and inflammatory mediators secreted by adipocytes initiate a negative feedback mechanism on the hypothalamic-pituitary (HP) axis. This feedback inhibits the secretion of gonadotropin-releasing hormone (GnRH) and subsequent release of luteinizing hormone (LH), ultimately resulting in diminished testosterone levels (46).

One of the strengths of this study lies in the thorough characterization of subjects based on a large population, and the use of subgroup analysis to evaluate changes in CMI and testosterone levels in different populations, thereby enhancing the credibility of the findings. Nevertheless, certain limitations still exist. Firstly, this trial only collected data from American male adults, which ignores the impact of CMI levels on testosterone levels in female adults and non-American populations, which is worth further exploration in the future. Secondly, the absence of gonadotropin data in NHANES database limits the ability to pinpoint the exact type of hypogonadism. Thirdly, the cross-sectional nature of this study precludes the establishment of causality, future research should consider using more longitudinal designs to deepen understanding of the research question. Fourthly, lack of data on testicular volume prevents its inclusion as a covariate in the study.

## Conclusion

In conclusion, CMI has an independent and negative correlation with testosterone levels in American male adults. Furthermore, CMI have higher AUC values of testosterone deficiency than WHtR and TG/HDL-C in American male adults. CMI provides a methodology for monitoring serum testosterone levels and recommends that patients experiencing testosterone deficiency address obesity and lipid levels. These findings hold substantial implications for clinical decision-making and preventive strategies.

## Data Availability

Publicly available datasets were analyzed in this study. This data can be found here: NHANES, www.cdc.gov/nchs/NHANES/.
